# Advances in Sonothrombolysis Techniques Using Piezoelectric Transducers

**DOI:** 10.3390/s20051288

**Published:** 2020-02-27

**Authors:** Leela Goel, Xiaoning Jiang

**Affiliations:** 1 Department of Mechanical and Aerospace Engineering, North Carolina State University, Raleigh, NC 27695-7910, USA; ldgoel@ncsu.edu; 2 Joint Department of Biomedical Engineering, North Carolina State University and The University of North Carolina at Chapel Hill, Raleigh, NC 27695-7910, USA

**Keywords:** sonothrombolysis, ultrasound enhanced thrombolysis, microbubbles, ultrasound contrast agents, high intensity focused ultrasound, histotripsy, intravascular ultrasound

## Abstract

One of the great advancements in the applications of piezoelectric materials is the application for therapeutic medical ultrasound for sonothrombolysis. Sonothrombolysis is a promising ultrasound based technique to treat blood clots compared to conventional thrombolytic treatments or mechanical thrombectomy. Recent clinical trials using transcranial Doppler ultrasound, microbubble mediated sonothrombolysis, and catheter directed sonothrombolysis have shown promise. However, these conventional sonothrombolysis techniques still pose clinical safety limitations, preventing their application for standard of care. Recent advances in sonothrombolysis techniques including targeted and drug loaded microbubbles, phase change nanodroplets, high intensity focused ultrasound, histotripsy, and improved intravascular transducers, address some of the limitations of conventional sonothrombolysis treatments. Here, we review the strengths and limitations of these latest pre-clincial advancements for sonothrombolysis and their potential to improve clinical blood clot treatments.

## 1. Background

Blood clots are a leading problem for health complications, with over 300,000 new cases of thrombi occurring in the United States annually [1,2]. One particularly dangerous category of blood clots are middle cerebral artery (MCA) occlusions, which often lead to stroke. The other blood clot of concern are deep vein thrombosis (DVT), which can greatly increase the risk of patients developing a pulmonary embolism and are a common source of post-surgery complications. Given the high incidence rates and serious complications which arise from different kinds of blood clots, rapid diagnosis and treatment are critical.

Two primary treatment approaches for blood clots are mechanical removal and thrombolytic drug treatments; however, these approaches have limitations. For mechanical removal of clots, there is a high risk of clot debris formation causing downstream pulmonary embolism and for endothelial vessel wall damage to occur, which can exacerbate an already pathological coagulation scheme in the body [3,4,5,6,7]. On the other hand, thrombolytic drug treatments such as tissue plasminogen activator (tPA) and urokinase (UK) are only effective for un-retracted blood clots and have to be administered within three hours of the onset of a stroke to treat MCA occlusions [3,8,9,10,11,12]. If these thrombolytic drugs are being used to treat deep vein thrombosis, those treatment times can last upwards of 15 h, making these treatment regiments taxing on patients both physically and increasing medical costs. Most notably, thrombolytic drugs can greatly increase the risk of hemorrhage in patients, potentially leading to intracranial hemorrhage or death [10,11,12]. In addition to systemic thrombolytic treatments, catheter based thrombolysis treatments are also used to help administer the thrombolytic agents directly to the clot of interest; however, these drugs are still ultimately circulated systemically, increasing the risk for non-intended clot lysis. Therefore, while these treatments are effective, further work needs to be done to mitigate the serious side effects of these approaches.

One of the great advancements in the applications of piezoelectric materials is the application for medical ultrasounds which have greatly improved patient diagnoses and resulting clinical outcomes over the last century. Given that ultrasound is a mechanical wave, piezoelectric materials are an excellent choice for both generating high amplitude ultrasound waves and also sensing low amplitude reflected sound waves in the body. Medical ultrasound devices typically use a piezoelectric ceramic material, most commonly a lead zirconate titanate (PZT) material. The element shape, number of elements, and aperture sizes depend on the application of the ultrasound transducer. Diagnostic and Doppler ultrasound, which take advantage of both the actuating and sensing capacities of piezoelectric materials being the current clinical standard of care for the diagnosis and monitoring of blood clots. More recently, piezoelectric materials have been applied for therapeutic ultrasound for medical uses. These applications include high intensity focused ultrasounds for non-invasive thermal ablation of tissue to using diagnostic ultrasound conditions for targeted drug delivery. Given that the clinical standard of care is using diagnostic and Doppler ultrasound for diagnosing blood clots and monitoring conventional clot treatment, it was a natural next step to utilize a medical ultrasound to aid with enhancing blood clot treatments.

**Figure 1:**
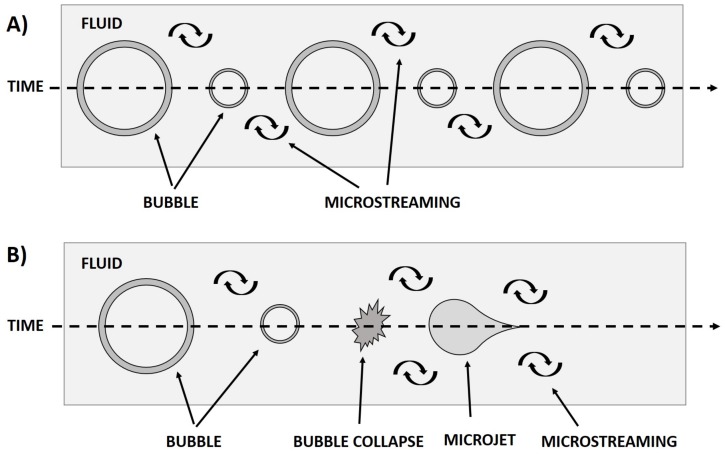
Schematic of (**A**) Stable Cavitation and (**B**) Inertial Cavitation. In this case, stable cavitation is the oscillation of a bubble or contrast agent, which causes fluid motion, or micostreaming. Inertial cavitation is the collapse of a bubble or contrast agent, resulting in both microstreaming and a stronger microjet of fluid.

Sonothrombolysis is the use of ultrasound to enhance thrombolysis treatment. The use of ultrasound enhanced thrombolysis (UET) has been observed since the late 1980s. Sonothrombolysis is typically applied to help enhance thrombolytic treatment outcomes. This treatment technique takes advantage of the mechanical bioeffects of ultrasound to aid in the diffusion of thrombolytic drugs into blood clots and to mechanically break up blood clots. The generally accepted mechanism of thrombolysis enhancement is that the ultrasound is able to induce stable cavitation, inertial cavitation, micro-streaming, and acoustic radiation force to temporarily ”loosen“ fibrin clots and increase diffusion of thrombolytic drugs into the blood clot, allowing for faster and more effective clot treatment (Figure 1) [13,14,15,16,17,18,19,20,21,22,23,24,25,26,27,28]. In addition to improving thrombolytic treatment, several studies and clinical trials have demonstrated that ultrasound contrast agents, namely microbubbles (MBs), can be used to enhance clot lysis with tPA by increasing the amount of targeted cavitation, thereby destroying the blood clots while minimizing clot debris [15,18,20,21,23,26,27,29,30,31,32].

### 1.1. Mechanisms

One commonly accepted mechanism of enhanced thrombolysis is by inducing clot displacement [17,19,20,22,23,26,28,29,31,33,34,35]. One of the main acoustic mechanisms of clot displacement is acoustic radiation force (ARF) [20,22,23,26,30,34]. Acoustic radiation force is dependent on the frequency dependent attenuation, acoustic intensity, and speed of sound of the medium, given by the equation for ARF. ($F_{ARF}=\frac{2\alpha I}{C}$). From here, we can notice in particular that changing the acoustic intensity and center frequency of the transducer would change the amount of force generated, and, by extension, the amount clot displacement. Therefore, it is important to consider the transducer design and acoustic parameters used in relation to the mechanisms of sonothrombolysis. Another widely accepted mechanism for sonothrombolysis is cavitation [13,14,15,16,17,18,19,20,21,22,23,24,25,26,27,28,33,36,37,38,39,40]. This includes both stable [16,21,22,23,26,41] and inertial cavitation [16,17,19,21,22,23,26,36,39]. Cavitation and displacement helps break up clots and aids in thrombolytic penetration, fibrin disaggregation, and acoustic streaming. Cavitation is thought to be a mechanism both with and without the presence of added contrast agents. In high intensity focused ultrasound (HIFU) and histotripsy, cavitation is able to be induced without contrast agents, while including microbubble contrast agents reduced the threshold necessary for cavitation. Cells within the blood clots may also act as nuclei for cavitation, resulting in additional clot destruction.

Acoustic streaming also facilitates improved thrombolytic outcomes [14,17,22,26,27,32,34,42,43]. Acoustic streaming hypothesized to help improve the penetration of thrombolytics and ultrasound contrast agents into the clot by both encouraging diffusion, transporting thrombolytic agents, and mechanical perturbation [15,18,20,21,23,26,27,29,30,31,32]. Another proposed mechanism is that ultrasound mediates redistribution of the clot surface thrombin and fibrin, allowing greater penetration into the clot [20,29,30,31,32,40]. It is also proposed that sonothrombolysis helps mediate fibrin disaggregation [20,21,26,28,32,44,45]. Other hypothesized mechanisms are biological effects such as the limitation of existing thrombin which is depleted from existing plasma, enzyme activation, and platelet activation by ultrasound [16,18,22,32,39,45,46,47,48,49,50]. While temperature was thought to potentially influence enzyme activity and increase diffusion, most studies have not found a major relationship between thermal mechanisms and thrombolytic outcomes [18,22,26,30,41,42,47,51,52].

The mechanisms of sonothrombolysis can be directly influenced by the acoustic parameters used by an ultrasound system from piezoelectric ultrasound transducers. While there have been a few studies directly examining the mechanisms of ultrasound enhanced thrombolysis, future work should be done to understand how the acoustic parameters chosen influence these mechanisms. However, while these mechanisms, particularly cavitation, have been shown to improve clot lysis outcomes, this can also lead to potentially dangerous bioeffects in the body which should be improved in future transducer design and insonation schemes.

### 1.2. Clinical Trials of Sonothrombolysis Techniques

Given the extensive amount of pre-clincial data on the potential effectiveness of sonothrombolysis for treating MCA occlusions, there have been several clinical trials using transcranial Doppler for MCA occlusion treatments with tPA [53,54,55]. While these trials have shown promise, the main limitation of this technique is the potential increased risk for patients to develop intracerebral hemorrhage. As such, further research should be done to help mitigate this risk from sonothrombolysis techniques.

There have also been clinical trials with transcranial Doppler to treat MCA occlusions with thrombolytic agents with the addition of microbubbles [56,57,58]. These trials have shown great promise in both improving thrombolysis outcomes and decreasing treatment time necessary for acceptable patient outcomes. While exciting, given the recent advances in targeted and drug loaded microbubbles and novel contrast agents, there is room for improvement to minimize the amount of thrombolytic drugs that patients need to receive in order to have positive outcomes, instead of simply adding more microbubbles to standard tPA doses.

Finally, there have been many clinical trials using the EKOS EndoWave system for catheter directed sonothrombolysis. However, it remains to be seen if this this side-looking, low power intravascular transducer improves overall patient outcomes compared to standard catheter directed treatments [59,60,61,62,63,64]. Therefore, it is important to examine alternative intravascular based sonothrombolysis techniques to improve clot lysis outcomes.

### 1.3. Purpose

Given the culmination of transcranial Doppler, microbubble mediated, and intravascular sonothrombolysis research to clinical trials, it is still necessary to improve these technologies to address the observed clinical outcomes and limitations. There have been several extensive reviews on the benefits of thrombolytic mediated sonothrombolysis, microbubble mediated sonothrombolysis, and intravascular sonothrombolysis [60,64,65,66,67,68,69,70,71,72,73,74,75,76,77,78,79,80,81]. However, there still exists a clinical need for thrombolysis techniques which can decrease the risk of hemhorrage, vessel damage, and pulmonary embolism while also reducing the dose of thrombolytic agents and treatment times for patients. The purpose of this review is to summarize the latest developments of sonothrombolysis techniques and their mechanisms which are still in the pre-clinical stages. We hope that, in examining the state of the art in sonothrombolysis techniques, further developments can be made to address the current clinical limitations of existing sonothrombolysis techniques.

In this review, we will examine the last decade of new approaches for sonothrombolysis treatments and address their potential benefits and limitations. First, the advances in novel contrast agents will be summarized including the use of targeted and drug-loaded microbubbles, phase change nanodroplets, and magnetic microbubbles. Next, the use of therapeutic ultrasound will be examined in for the cases of high intensity focused ultrasound, histotrispy, and microtripsy. Finally, improvements for intravascular and catheter directed sonothrombolysis will be summarized. By examining the latest advances in sonothrombolysis treatments, we may develop approaches for improving patient outcomes compared to traditional thrombolysis treatments.

## 2. Novel Contrast Agents for Sonothrombolysis

**Figure 2:**
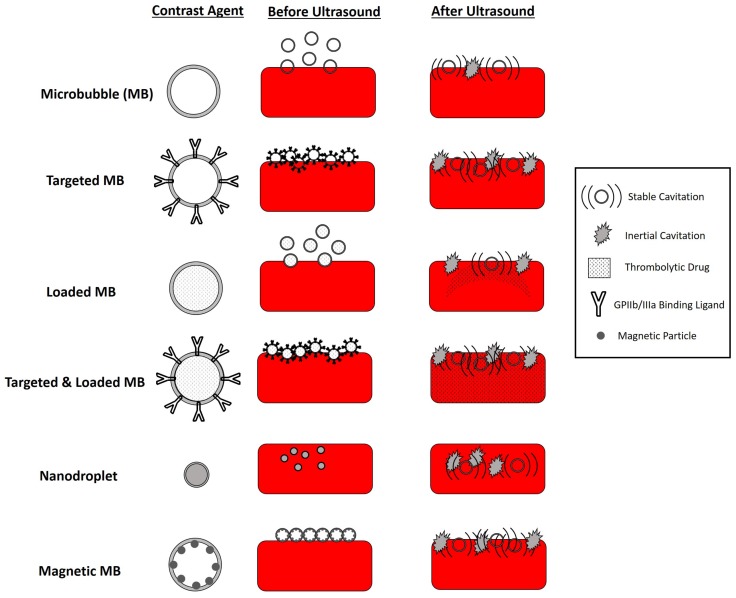
Schematic of Novel Contrast Agents for Sonothrombolysis.

The use of standard lipid shelled microbubbles such as Sonovue and Definity either alone or in combination with thrombolytic agents such as tPA have been studied and reviewed in great detail [67,75]. Microbubbles both alone and when used in combination with a thrombolytic agent for sonothrombolysis treatment uniformly outperform traditional thrombolytic treatments. However, the limitations of microbubble circulation time, microbubble concentration at the clot surface, and ability to reduce thrombolytic agent dose remain to be addressed. There have been some exciting advancements of using targeted microbubbles, drug loaded microbubbles, nanodroplets, and magnetic microbubbles to aid in sonothrombolysis to address the limitations of traditional MB mediated sonothrombolysis. The summarized acoustic parameters for these techniques and schematics can be found in Table 1 and Figure 2, respectively. These parameters are important to keep in mind as they can influence the thrombolytic efficacy of these devices. Contrast agent mediated sonothrombolysis has been applied for treating middle cerebral artery occlusions, pulmonary embolisms, and deep vein thromboses due to their ability to travel systemically and improve targeted drug delivery.

Targeted microubbles can be defined as microbubbles which selectively bind to specific tissue types. In this case, researchers have developed microbubbles which can selectively bind to glycoprotein IIb/IIIa (GPIIb/IIIa), found in the activated platelets which make up thrombi [45,82,83,84,85,86]. The benefit of this is that, compared with non-specific microbubbles, when activated by ultrasound to induce stable and inertial cavitation, these effects will only affect the thrombus of interest while minimizing the potential risks of vessel damage to the non-targeted thrombus. This would allow greater treatment of the clot itself and, if used in combination with a thrombolytic drug, allow more of that drug to penetrate the thrombus. In in vivo canine studies and rat models, targeted microbubbles have demonstrated significant improvements in sonothrombolysis outcomes compared to their non-targeted microbubble counterparts.

Drug loaded microbubbles are of interest due to their potential to minimize non-site specific tPA or urokinase binding. Because the thrombolytic drug is primarily released when activated by ultrasound, and the ultrasound is only focused on the clot of interest, this may both minimize hemorrhage risks while also taking advantage of the synergistic improvements of thrombolysis outcomes with MBs and thrombolytic agents [87,88,89,90,91,92]. Both urokinase loaded echogenic liposomes and tPA loaded echogenic liposomes outperform using a thrombolytic agent alone or thrombolytic agent mediated sonothrombolysis. Interestingly, these drug loaded liposomes exhibit similar clot lysis outcomes compared to treatments of microbubbles alongside thrombolytic agent treatment [87,89]. Additionally, there is evidence that a lower UK dose can be used to still be effective for clot lysis outcomes when using drug loaded liposomes [89]. These outcomes are important because it provides evidence that it may be possible to reduce the total exposure of thrombolytic agents to the patient, thus reducing the likelihood of side-effects.

There has also been recent work done to combine the benefits of both targeted and loaded microbubbles to further enhance sonothrombolysis treatments [93,94,95]. These preliminary studies have demonstrated that these loaded and targeted microbubbles outperform thrombolytic mediated sonothrombolysis and traditional microbubble mediated sonothrombolysis. Given the recent studies on targeted, loaded, and targeted and loaded microbubbles, a wide variety of both custom and commercial transducers and parameters have been used. It will be important to examine the effects of these parameters on the mechanisms of thrombolysis, thrombolytic outcomes, and safety.

Phase change nanodroplets are also of recent interest given the much smaller initial size of these nanodroplets compared to microbubbles [36,96,97]. This may help provide better spatial control to minimize potential for vessel damage, increase generated pores in the clot of interest to improve diffusion of thrombolytic agents into the clot, and generate smaller clot debris with this technique. This technique has been implemented with both high intensity focused ultrasound and diagnostic ultrasound transducers, making it important to assess the potential benefits of different types of therapeutic ultrasound for this technique. However, further work needs to be done to help ensure the safety of nanodroplet mediated sonothrombolysis and verify these potential clinical benefits.

One of the limitations of traditional microbubble mediated sonothrombolysis is the ability to target the microbubbles to stay in the clot region of interest and minimize circulation of microbubbles to other parts of the body. Magnetic microbubbles (MMBs) are able to be guided using a magnet to allow accumulation of MMBs in the region of interest [98]. Then, upon insonation with ultrasound and a magnetic field, these bubbles are able to induce stable and inertial cavitation, thus inducing sonothrombolysis. Some in vitro studies have demonstrated the feasibility of this technique in vitro for increasing the local microbubble concentration at a thrombus and for improving clot lysis outcomes compared to non-magnetic microbubbles [99,100]. While promising, work needs to be done to assess the toxicity of magnetic microbubbles as well as the feasibility of implementing MMBs in current clinical settings given that only custom sub-megahertz transducers have been developed for this technique.

These advances in ultrasound contrast agents for sonothrombolysis applications have the potential to improve localized MB concentration, improve targeted drug deliver, and minimize patient exposure to off-target thrombolytic agent exposure. In addition to modifying the insonation parameters, it will be important to improve transducer design to improve lytic efficiency and and safety. Future work should be done to evaluate the ability to minimize the dose and side effects associated with thrombolytic agents. Additionally, the safety effects of all of these microbubble mediated techniques should be evaluated to minimize vessel damage, hemorrhage, and potential for pulmonary embolism.

## 3. Therapeutic Ultrasound

**Figure 3:**
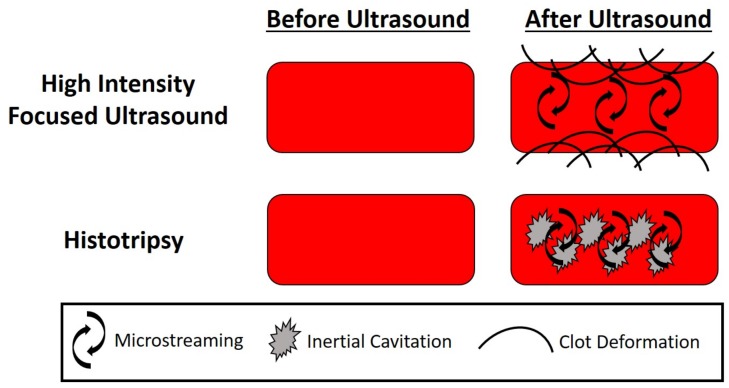
Schematic of Therapeutic Ultrasound for Sonothrombolysis.

One of the major appeals of sonothrombolysis is the potential to eliminate or greatly reduce the need for a thrombolytic agent for clot lysis. The use of therapeutic ultrasound techniques such as high intensity focused ultrasound and histotripsy have demonstrated the potential to eliminate the need for thrombolytic agents, and are summarized below. The acoustic parameters for these techniques and schematics are shown in Table 2 and Figure 3, respectively. Because these techniques typically do not utilize ultrasound contrast agents, they tend to use much higher peak negative pressures and power outputs in order to generate cavitation and acoustic radiation force compared to contrast agent mediated sonothrombolysis.

High intensity focused ultrasound (HIFU) has had great success for therapeutic ultrasound applications, particularly in transcranial applications [101,102]. While traditional HIFU treatments induce thermal ablation for tissue destruction, in sonothrombolysis applications, a pulsed ultrasound scheme is used which is below the thermal ablation threshold and, instead, the primary mechanism of clot lysis is acoustic radiation force and localized tissue displacements and clot deformation [29,33,103]. Early studies using HIFU for sonothrombolysis demonstrated that HIFU can enhance tPA mediated sonothrombolysis [29,103]. However, more recent studies have demonstrated the ability of HIFU to induce clot lysis without the need of a thrombolytic agent in both in vitro and in vivo animal studies [33,44,104,105,106,107,108,109,110]. Researchers have demonstrated that shorter pulse repetition frequencies, higher duty cycles, and higher peak negative pressures increase clot lysis results [33,107]. Both custom ultrasound transducers and commercial HIFU devices have been utilized for pre-clinical sonothrombolysis studies. While reducing the need for a thrombolytic agent can mitigate the potential for hemorrhage from a thrombolytic, the potential for vessel damage and pulmonary embolism should be assessed. Several in vitro studies have demonstrated the clot debris produced from HIFU are small enough to mitigate the risk of pulmonary embolism [33,104]. However, in in vivo animal studies, there has been some evidence of potential hemorrhage due to vessel damage, potentially due to standing waves generated [103,105]. One hypothesis to mitigate the risks of damage due to standing waves and high intensity ultrasound is to use dual-frequency HIFU and incorporate dual-frequency HIFU with contrast agents to reduce the acoustic pressure necessary to generate lysis [108,109,110]. Recent work has demonstrated the dual-frequency HIFU may reduce the acoustic energy necessary for sonothrombolysis outcomes. However, further in vivo assessments should be done for HIFU mediated sonothrombolysis to evaluate the potential risks for pulmonary embolism, vessel damage, and hemorrhage.

Histotripsy is a non-thermal ablation method which has been successful in tissue destruction via induced cavitation, and recently it has been applied for sonothrombolysis. One attractive feature of histotripsy is the ability to fractionate clots without the need of thrombolytic agent, thus eliminating the associated risks of thrombolytic treatments [38,39,40,111,112,113,114]. Early histotripsy studies demonstrated that this technique was effective for destroying clots and that the resulting clot debris can be minimized by adjusting the peak negative pressure, duty cycle, and pulse repetition frequency of the pulses [39,40,111]. However, while clot debris sizes were primarily limited to less than 100 μm, initial in vivo animal studies demonstrated some vessel damage and hemorrhage in the region of excitation [111]. To address this, researchers developed a technique termed microtripsy, which allows the benefits of histotripsy while benefiting from a much smaller focal region, thus minimizing the risk for vasculature damage [38,112,113]. In in vivo animal studies, while some vessel damage and hemorrhage were observed, there was no damage observed in two weeks follow-up, indicating that this technique may improve the previous histotripsy technique [113]. Unlike other sonothrombolysis techniques, initial in vitro studies have indicated that histotripsy may be used to treat retracted clot models, which has implications for using sonothrombolysis to effectively treat deep vein thromboses [38,114]. Additionally, while the clot debris sizes for all histotripsy techniques were theoretically low (less than 1000 μm as to minimize the risk for pulmonary embolism, further animal studies should be done to verify.

Both HIFU and histotripsy have traditionally been applied for non-invasive tissue destruction techniques. While HIFU has typically been applied for transcranial blood clots using traditional HIFU transducer systems, new transcutaneaous designs may allow for the treatment of deep vein thromboses. The histotripsy transducer is a transcutaneous device, allowing it to be best applied for deep vein thromboses models. It will be imperative to ensure that therapeutic ultrasound mediated sonothrombolysis will be able to minimize unintended bioeffects and tissue damage that do not exceed conventional mechanical treatments for blood clots while still maintaining clinically acceptable clot lysis outcomes.

## 4. Intravascular Sonothrombolysis

Intravascular sonothrombolysis is an attractive technique due to the potential for site specific ultrasound insonation which greatly minimizes the likelihood of vessel wall damage or unintended bioeffects. Given the benefits of catheter directed thrombolytic therapy, using ultrasound in conjunction with catheter directed therapy should improve sonothrombolytic outcomes. Currently, the EKOS EndoWave system is being using in clinical trials for catheter directed sonothrombolysis for many types of thrombi including pulmonary embolisms and deep vein thromboses; however, it remains to be seen if this system improves overall patient outcomes compared to conventional treatments [64]. However, there have been some recent developments in using catheter directed sonothrombolysis by utilizing ultrasound contrast agents and designing new transducers.

Two recent in vitro studies have utilized various intravascular sonothrombolysis techniques mediated with microbubbles. One study again uses the EKOS system, which is a side-viewing low power, high frequency transducer, and demonstrates that this device is able to improve clot lysis outcomes with tPA and microbubble mediated sonothrombolysis in an in vitro deep vein thrombosis model [115]. Another study from our research group developed a forward viewing, sub-megahertz intravascular transducer for sonothrombolysis treatment which aims to be applied for deep vein thrombosis [116]. These parameters were optimized for microbubble mediated sonothrombolysis without the need for a thrombolytic agent, and in vitro studies have shown great success in treating blood clots with this technique.

While there has been some developments to address the clinical limitations of existing catheter directed sonothrombolysis techniques, further work needs to be done in this area to optimize intravascular sonothromboysis for tPA mediated treatment, and to assess the safety of microbubble mediated sonothrombolysis to minimize vessel damage and pulmonary embolism.

## 5. Conclusions and Future Directions

Recent advances in sonothrombolysis techniques have addressed some of the major limitations of conventional blood clot treatment including reduction of thrombolytic agent drug exposure, minimizing the risks of pulmonary embolism, and decreasing the potential for hemorrhage. Given that these techniques are primarily still in the early pre-clinical stages, there exists a need to assess the potential side-effects and side-effects of novel sonothrombolysis approaches in an in vivo animal setting in order to verify improvements to conventional blood clot treatments. It is also worth applying the techniques of modifying the duty cycle, peak negative pressure, and pulse repetition frequencies of existing Doppler ultrasound mediated sonothrombolysis techniques to see if standard microbubble mediated sonothrombolysis and thrombolytic agent mediated sonothrombolysis may be improved with existing technology. Improving insonation schemes using clinical ultrasound systems may make clinical translation more practical by potentially allowing clinicians to use their existing ultrasound systems.

Exposure time, frequency, acoustic intensity, acoustic power, and duty cycle influence clot lysis results. All of these parameters are able to influence ARF and cavitation potential in the tissues. While methods such as histotripsy and microbubble mediated sonothrombolysis optimize parameters to induce cavitation, others such as HIFU optimize for higher ARF forces and displacements to facilitate microstreaming. However, further studies should be done to directly examine the influence of these parameters on thrombolytic outcomes and elucidate the exact mechanism of ultrasound enhanced thrombolysis. Elucidating the exact mechanisms for sonothrombolysis may help drive future transducer development to optimize for certain bioeffects that are most effective for a given clot type of interest. Work also needs to be done to standardize the reported parameters and measures in order to compare studies and evaluate the influence of given parameters on lysis outcomes.

It is important to keep in mind the biological/animal model [117], thrombolytic agent, [14,18,118,119], and blood clot age [16,46,52,119,120,121] used in pre-clinical studies. The same acoustic parameters may result in different thrombolytic outcomes given different physiological models. Future directions should include considering combining different sonothrombolysis approaches to optimally minimize thrombolytic agent dose, vessel damage, clot debris size, and treatment times while improving overall patient outcomes. Additionally, further work should be done to apply sonothrombolysis techniques for treating deep vein thromboses, which are currently difficult to treat using conventional thrombolytic therapies given the older, retracted clots compared to middle cerebral artery occlusions which tend to be fresh, un-retracted clots.

Before moving on to clinical trials, it is imperative that the ultrasound parameters themselves are examined and understood in an in vitro setting. These parameters and transducer design should be used to minimize negative bioeffects while maximizing thrombolytic outcomes. Understanding these ultrasound parameters can help guide advances in sonothrombolytic techniques and potentially combine different approaches for sonothrombolysis. It is also important to consider other non-acoustic factors such as thrombolytic agent, animal model, and disease of interest when developing new sonothrombolysis techniques.

## Figures and Tables

**Table 1:** Ultrasound parameters for novel contrast agents.

Contrast Agent	Ref.	Transducer	Cent. Freq.	Intensity (W/cm^2^)	Duty Cycle (%)	PRF	MI
Targeted MBs	[45,82,83,84,86]	Therapeutic US Device (Dongjian Company, Wuhan, China); V302 Panametrics-NDT (Olympus Corp., Tokyo, Japan); P4-2, Philips HDI 5000 (Philips, Amsterdam, The Netherlands); Timi3 Systems (Timi3 Systems, Santa Clara, CA, USA); Custom prototype 10 PZT disks (Fuji Ceramics, Tokyo, Japan)	27 kHz–2 MHz	1.4–1.2	5–50	25–150 Hz	1.2–3.2
Loaded MBs	[87,88,89,90,91,92]	Custom H160 single element transducer (Sonic Concepts, Inc., Woodburn, WA, USA); Therapeutic US Device (Dongjian Company, Peijing, China); 8L-RS, Vivid i (GE Medical Systems, Milwaukee, WI, USA)	120 kHz–5.7 MHz	0.5–2.79	50–100	1.7–5 kHz	-
Targeted and Loaded MBs	[93,94,95]	CSY-2 (Puji, Chongqing, China); LA240 (Yum Mylab 90, Yum Mylab, Italy)	1.6–2.8 MHz	1.8	95	-	1.4
Magnetic MBs	[99,100]	Custom single element transducer (Sonic Concepts, Inc., Woodburn, WA, USA); Custom prototype 6 PZT-5A thin plates	500–620 kHz	-	1.6–10	0.2 Hz	-

Abbreviations: PRF—Pulse Repetition Frequency, MI—Mechanical Index, MBs—Microbubbles.

**Table 2:** Ultrasound Parameters for Therapeutic Ultrasound.

Therapeutic Ultrasound Technique	Ref.	Transducer	Center Freq.	Power (W)	Duty Cycle (%)	PRF	PNP (MPa)
High Intensity Focused Ultrasound	[33,44,104,105,106,107,108,109,110]	ExAblate 4000 High Intensity Focused Ultrasound headsystem (InSightec, Inc, Tirat Carmel, Israel); H-102 (Sonic Concepts, Bothell, WA, USA); Custom transducer (Blatek, Inc., State College, PA, USA)	220 kHz–1.51 MHz	0–550	2.5–50	1–1000 Hz	1.2–3.2
Histotripsy & Microtripsy	[38,39,40,111,112,113,114]	Spherical focused transducer, 18-element therapy transducer (Imasonic, Besancon, France)	1–1.2 MHz	-	0.1–18	5–1000 Hz	2–35

Abbreviations: PRF—Pulse Repetition Frequency, PNP—Peak Negative Pressure.
